# Improving access to preparatory information for children undergoing general anaesthesia for tooth extraction and their families: study protocol for a Phase III randomized controlled trial

**DOI:** 10.1186/1745-6215-15-219

**Published:** 2014-06-11

**Authors:** Marie Therese Hosey, Ana Nora Donaldson, Corinne Huntington, Christina Liossi, Patricia A Reynolds, Reham Alharatani, J Timothy Newton

**Affiliations:** 1 King’s College London Dental Institute, Bessemer Road, London SE5 9RS, UK; 2 Department of Psychology, University of Southampton, Building 44 Highfield Campus, Southampton SO17 1BJ, UK

**Keywords:** General anaesthesia, Children, Preparatory information, Coping, Cognitive behavioural therapy, Role modelling, Early childhood caries, Dental anxiety, Randomized controlled trial (RCT)

## Abstract

**Background:**

Children can find anaesthesia induction especially distressing and postoperative psychological and physical morbidity are common. Preparation programmes for general anaesthesia (GA) are highly effective in reducing this distress. A Phase II study has already verified the effectiveness of a prototype preoperative GA-coping computer game to help children cope with induction in a dental GA setting. The biggest patient users of pediatric GA services in the UK are children who need to have teeth removed (estimated to be 100,000 yearly). Tooth decay is the most common disease in children worldwide. This study is a Phase III randomized controlled trial (RCT) and will evaluate the effectiveness of the new internet version of this game.

**Methods/design:**

The Phase III RCT will use a double-blind three-armed design. The clinical trial will recruit up to 210 children and will compare the web-based game against standard care and another non-medical game. At least 53 patients in each group will be required for 90% statistical power. Distress will be assessed through an evaluation of the child’s behaviour during the visit and later parental reports of physical and psychological morbidity. The satisfaction of parents and children will be measured; the mode of usage of the web-based game will be automatically recorded and the impact on the service (for example, recovery time and throughput) will be reported.

The Phase III study primary outcome will measure: (1) patient experience: acceptance of anaesthetic induction, child cooperation and distress, reduction of peri- and postoperative morbidity, child and family satisfaction, and (2) service improvement: anaesthetic time and improvement in throughput. Measures will be administered at baseline, at the time of the GA treatment visit, and at 48 hours and one week postoperatively.

**Discussion:**

This study aims to determine the effectiveness of an online GA-coping game for children and families undergoing tooth extraction under GA.

**Trial registration:**

ISRCTN18265148 (registered 24 November 2013).

## Background

For a child, the induction of general anaesthesia (GA) appears to be the most stressful part of the surgical visit [1-7]. Psychological preparation of children beforehand has been shown to be highly effective in reducing distress and postoperative complications [8]. Ideally, preparation should be provided *at least* 5 days in advance for children aged 6 years and over and no more than a week in advance for children younger than this [9].

The use of computers and web-based psychological intervention is a growing area of interest with the notable advantages of accessibility and engagement at the individual’s own pace [10]. The use of computer-supported cognitive behavioral therapies has been recognized as a level I form of intervention in the ‘Improving Access to Psychological Therapies’ framework [11]. Our previous patient and public involvement work confirmed that over 70% of these families had computer access and internet capability to enable software such as ‘YouTube’ and they indicated that they would welcome this type of intervention.

Tooth decay is the most common disease in children worldwide [12]. The UK has the highest rate of pediatric GA for tooth removal in Europe, estimated at 100,000 per year (one of the highest in the world) and this is the most common reason for paediatric hospital admissions for GA. The children are commonly around 6 years of age and physical and psychological morbidity at 24 hours and 7 days has been reported as 63% and 24%, respectively. Behavioural disturbances include bad dreams, crying, disobedience, separation anxiety, temper tantrums, and bed-wetting [13,14].

A Phase II randomized controlled trial (RCT) provided proof of the concept that preparatory information helped to facilitate coping by means of a computer game in children undergoing tooth extraction. That study has been included in a 2010 Cochrane review but the game was dated, not in a format suitable to go online, and relied upon delivery at the time of the GA visit (just prior to surgery), and so has never been able to be made widely available [15,16]. The aim of this paper is to present the methodology of a Phase III RCT to test the efficacy of an updated version of the prototype, available online at home, to facilitate coping in 5 to 7-year-old children undergoing GA for dental extractions. The game includes simple cartoons with animation and a degree of interactivity with two imbedded role-modelling videos. It shows a child coping with the GA visit to undergo tooth extraction, later recovery, and finishes with family oral health messages. This is a similar template to the version used in the Phase II RCT [13].

## Methods/Design

### Primary aim

To evaluate the effectiveness and usability of an online GA-coping game to deliver preparatory information to families of children scheduled for GA by conducting a Phase III RCT in respect to: (1) patient experience: acceptance of anaesthetic induction, child cooperation and distress, reduction of peri- and postoperative morbidity, child and family satisfaction, and (2) service improvement: anaesthetic time and improvement in throughput.

### Secondary aims

The secondary aims are as follows: to report on the usage, accessibility, and acceptability of this type of interactive resource; and to better understand how families interact with online health information.

### Research question

This study poses two research questions. Firstly, would a web-based GA-coping game containing psychological preparatory information help children scheduled for general anaesthesia cope better with the event? Secondly, would this improve patient experience and satisfaction and enable improved service throughput?

### Null hypothesis

The null hypothesis is that the GA-coping game will not improve the child’s coping behaviours at anaesthetic induction and the parents will report neither improvements in the quality of information received nor benefits in respect to physical or psychological behaviour at the time of the surgical experience, or afterwards. Children and families will not report improved access to information about GA or ease of access or satisfaction attributable to web-based information.

### Plan of investigation

#### Pilot study

The pilot study aims to: (1) estimate recruitment rate by confirming inclusion and exclusion criteria and willingness of potential recruits to take part; (2) calibrate the blinded observer’s (RA) scoring of the Visual Analogue Scale (A-VAS) at the time of GA induction and train in taking the other measures such as the modified Yale Preoperative Anxiety Scale (m-YPAS); (3) determine the best way to enable blinding both of the participants and the blinded researcher on the ward; (4) avoidance of the ‘Hawthorne effect’; (5) test the randomization process; and (6) train in video camera data capture [17-19].

### Pilot study method/design

The pilot study followed the same design and recruitment criteria and method as the planned Phase III RCT, with the only difference being that children were only assigned to one group. In order to mimic the actual protocol of the main study, participating children were given a two-sided colouring sheet at the pre-assessment clinic to take home. Their compliance was checked preoperatively on the day of the surgery by asking the children if they had coloured the pages.

### Researcher training

The blinded observer (RA) underwent a six-week training period that included rehearsing the protocol, becoming familiar with the Day Surgery Unit and GA service, completion of observational measures and, most importantly, practising ‘going unnoticed’.

### Pilot study results

**Figure 1:**
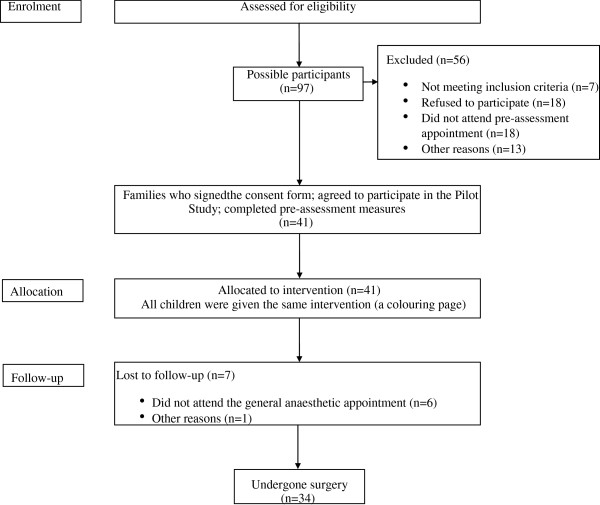
Pilot study flow diagram.

The pilot study was undertaken over a three-month period, linked to blinded observer training. A total of 41 children and families volunteered and consented to participate. There was complete data for 34 families, 56% of children were 6 year-olds; 68% were males and 32% were females. Further detail is shown in Figure 1.

With regards to dental anxiety, the mean Modified Child Dental Anxiety score (MCDAs) was 24 (range 12 to 40), and 53.5% of the children were ‘dentally anxious’ (scores above 18) [20]. Furthermore, child-reported preoperative anxiety using the Facial Image Scale (FIS) on the ward confirmed that 41.5% of the children were fairly to extremely worried at this time [21]. The mean A-VAS at induction was 2.6 (range 0 to 10) (0 = calm or no distress; 10 = highly distressed) with a median of 7 teeth extracted (range 2 to 16). Intravenous paracetamol during the surgery was given to 73.2% of the children.

### Eligibility criteria

It was a challenge to determine the fluency of the families’ spoken English and whether a child had a diagnosed learning difficulty until after they had already agreed to take part in the trial. If the child’s family had any previous experience of GA they were originally planned to be excluded from the Phase III RCT, bearing in mind that these families have a rate of 47% repeat dental GA experience [22]. However, it became apparent that some children had undergone surgery as babies (for example, during circumcision). Since this was unlikely to be remembered by them, the eligibility for the main study was relaxed to include those who had had prior surgery under the age of two years.

### Blinding and observer training and calibration of anaesthetic induction measures (VAS and m-YPAS)

Behaviour at induction was scored using a 10 cm VAS [16] for each participant, this is labeled as the A-VAS to differentiate from other usages of a visual analogue scale in the study. In order to calibrate the anaesthetic induction measures, a random selection of the pilot videos was viewed and the scores compared to the unblinded observer scores (CH); Cohen’s kappa confirmed that the level of inter-observer agreement was **κ** = 0.60, which is substantial. The blinded observer (RA) later rescored the videos; Cohen’s Kappa confirmed that the level of intra-observer agreement was **κ** = 0.80, which is substantial to almost perfect. Therefore, for the RCT, the blinded observer (RA) will use direct observation to score the A-VAS at the time of anaesthetic induction as well as capturing the event on video to enable future assessment of reproducibility and observer agreement.

### m-YPAS

Originally, this measure was planned to be taken at only one time point, the preoperative holding area (the ward). However, it was noticed that the children’s anxiety state changed as soon as they were told they were next in the list order to go into the anaesthetic induction room. Moreover, in their original article validating the m-YPAS, Kain *et al*. advised taking the measure at two points in time [17]. Therefore, timing for the m-YPAS was adjusted for the RCT to include (a) on the ward, just prior to summons to the induction room (m-YPAS-Time1) and then again (b) on entry into the induction room (m-YPAS-Time2). Due to the fast throughput of this service it was clear that the blinded observer (RA) would not always be able to capture the m-YPAS-Time1 if they were already in the operating theatre collecting data from a preceding patient. Although this might be a rare occurrence, the unblinded observer (CH) might have to score this measure. Therefore, the two researchers (CH and RA) trained together and achieved inter-observer and intra-observer reliability on the m-YPAS scores during the pilot **κ** = 0.93 to 0.96.

### Randomization method

The procedure for dynamic randomization was developed during the pilot to ensure that all three groups were matched in terms of age and gender. To achieve allocation concealment and to avoid selection bias, the unblinded research associate (Corinne Huntington) prescreened case notes to estimate possible numbers of recruits and their age and gender in advance of the once weekly recruitment capture point. The research statistician gave them the allocation. Participant folders were made up in advance which were visually identical irrespective of group allocation but the contents inside varied. The unblinded researcher (CH) used the pilot study to practice the invitation, recruitment, randomization, allocation, and delivery of these packs to enhance recruitment rate and to ensure participant blinding.

### Researcher blinding and avoidance of the Hawthorne effect

The researchers developed processes that were then used in the RCT to go undetected by staff and participants and to avoid interfering with the hospital team’s daily workflow or the patient’s GA process. A perioperative blind data collection procedure was developed using colour-coded data sheets. The blinded observer (RA) always remained in the background and was never introduced to participants. The hospital team acclimatized to her presence and normalized their work. This was to avoid the Hawthorne effect as best as was possible [19]. The unblinded researcher took care to avoid unmasking the blinded observer.

Because many of the Day Surgery Unit (DSU) staff were interested in learning about the study, a sample packet of the measures was created for them to see. The DSU team became familiar with the study and data collection process and their behaviour became normalized. Moreover, they did not realize that the early participants were in the pilot study only and they were not informed of the actual start of the Phase III RCT data capture.

### Video recording

During the pilot study, most of the anaesthetists were ‘camera shy’ and were hesitant to comply with video recording of the induction process even though the participants had agreed to be filmed. Meetings were held with them to inform them about the RCT and they were invited to attend grant team meetings. As a result of this, the anaesthetists themselves suggested the best and least obtrusive placement of the video camera for the RCT. In addition, the anaesthetists themselves scored the ease of induction, which helped to avoid them accidentally asking the child about the intervention used and thus unblinding the blinded observer (RA). Ultimately, if any anaesthetist does not agree to recording, the A-VAS score used in the RCT will be from the direct observation of the blinded observer (RA).

### Familiarity with hospital procedures

The researchers (CH and RA) developed methods, for use in the RCT to extract relevant hospital computer system information regarding patient throughput.

### Parental satisfaction

Parents’ feedback during the pilot led to three changes to the final method of capturing the patient satisfaction measures in the RCT: (1) the Dental Visit Satisfaction Scale questionnaire was confusing, irrelevant to the GA visit, and difficult to answer, and so this was removed from the RCT protocol; (2) the satisfaction measure at discharge was simplified to include only questions about satisfaction with the service and the preparatory information received, and these were also scored using a 10 cm VAS, we have annotated this as the S-VAS to differentiate from the 10 cm VAS used at anaesthetic induction (A-VAS) (3) the treatment evaluation inventory (TEI) was modified to give TEI ‘overall’ and TEI for ‘preparatory information’ scores [23].

### Summary of pilot

The pilot study refined the RCT and developed standard procedures for recruitment, randomization, blinding, and data capture and led to the modification of some of the measures and the time points at which they were collected. The methodology of the Phase III RCT is complex and as a result, the pilot explored the implementation and setting and ensured that the protocol was feasible in a busy hospital Day Surgery Unit whilst ensuring appropriate and consistent measures [24].

#### Phase III RCT

##### Methods and design

**Figure 2:**
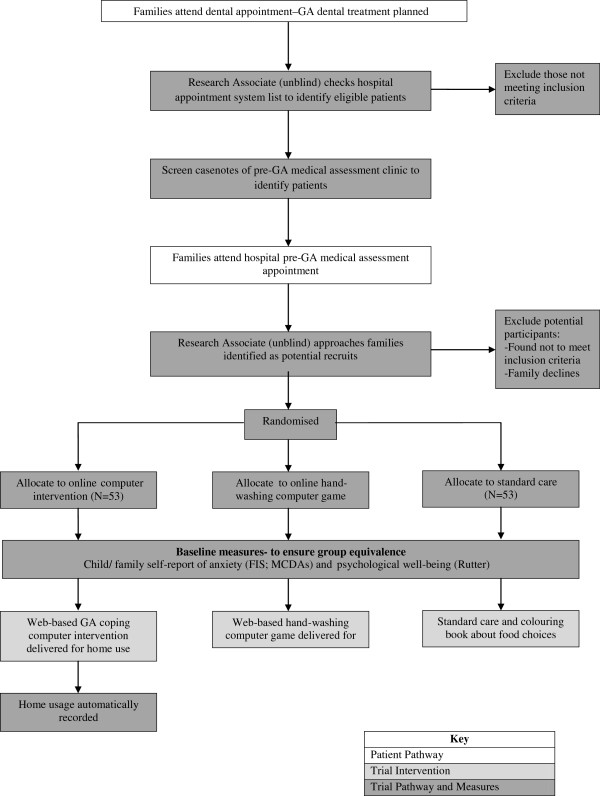
**Phase III RCT: flow diagram showing recruitment, randomization, and group equivalence measures.** For information on the Rutter Scale see [25]. FIS, Facial Image Scale; GA, general anaesthetic; MCDAs, Modified Child Dental Anxiety score.

**Figure 3:**
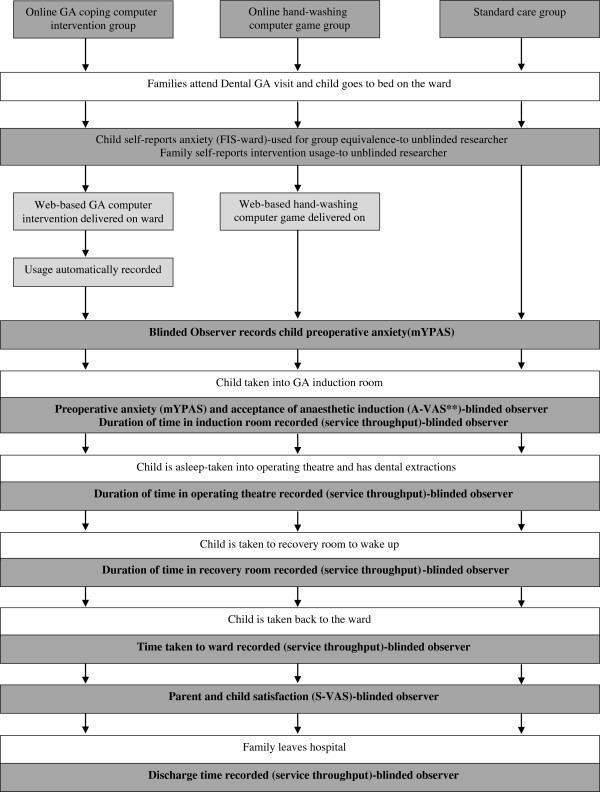
**Phase III RCT: flow diagram showing key measures at the GA dental visit.** **90% power calculation measure. A-VAS, 10 cm Visual Analogue Scale at anaesthetic induction; GA, general anaesthetic; m-YPAS, modified Yale preoperative anxiety scale; RCT, randomized controlled trial; S-VAS, 10 cm Visual Analogue Scale used to score patient satisfaction.

**Figure 4:**
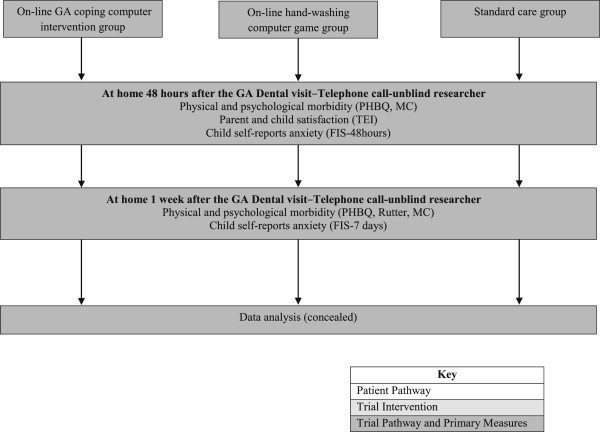
**Preparatory information for children undergoing GA for tooth extraction: flow diagram showing key measures postoperatively.** For information on the Rutter Scale see [25]. FIS, Facial Image Scale; GA, general anesthesia; MC, Morbidity Checklist, PHBQ; the Post Hospital Behavior Questionnaire; TEI, Treatment Evaluation Inventory.

The design is a double-blind randomized controlled trial to evaluate the online GA-coping game. Participants will be randomized into one of three groups: (1) Study Group: the online GA-coping game plus standard care (a leaflet containing fasting instructions); (2) Blank Control Group: standard care plus a colouring book (about health food choices); and (3) Computer Control Group: standard care plus an online non-medical computer game (about hand-washing). It is routine practice for patients to use hand gel on entry to the Day Surgery Unit as part of the normal hospital infection control policy. This control group hand-washing computer game is of a similar length and level of interactivity as the intervention but, unlike the intervention, has no psychological support regarding coping with the hospital visit, information about tooth extraction, or dental prevention information. It will act as a control against the possible effect of computer usage causing a distraction to the child whilst on the ward. The design is shown in Figures 2, 3 and 4.

### Ethical considerations

Ethical approval was obtained through the South East London Research Ethics Committee 2 (reference number: 10/H0802/41). Informed consent will be sought from each participating parent or guardian and assent will be sought from the children themselves. Participants will be able to withdraw from the trial at any time and this will not affect access to the GA treatment. It was the ethics committee who asked for inclusion of the colouring book in the standard care control group. The researchers have agreed to do so as this may help blind the participants.

All information disclosed in the study will be kept confidential and participants will not be identifiable in any material published as part of the study in any way. All data are stored without any identifying details under secure conditions and all videos will be destroyed at the end of the trial. An independent Data Monitoring Committee will be convened and the reporting of serious adverse events will be made to the chief investigator. The chief investigator will then inform the research and development manager. Local policy and procedures will be followed for reporting and investigating serious adverse events.

### Participants

Up to a total of 210 participants will be recruited and allocated at random to one of the three groups. This will allow for attrition between recruitment and the GA dental visit as well as the home follow-up data capture time points. Participants will be 5 to 7-year-old children (representative of the common age range in children undergoing this procedure in the UK) and their carers, for whom it has already been determined that dental extraction under GA is required. This cohort of patients usually (but not exclusively) receives a gaseous induction of anaesthesia (inhalation of the anaesthetic gas through a face mask).

The participants will be recruited at the time of their pre-GA medical assessment in King’s College Hospital Day Surgery Unit. This clinic occurs once a week and families are scheduled to attend approximately two weeks in advance of their GA dental visit. This is a nurse-led medical check to confirm fitness for GA. There are approximately 1,000 children scheduled for this treatment each year.

The inclusion criteria are as follows: children must be between 5 and 7-years-old, parental consent and child assent must be given to participate, must be literate in English, must own or have access to a computer with internet access, have no previous experience of GA for dental reasons, and have no previous experience of GA after the age of 24 months.

The exclusion criteria are as follows: any prior experience of dental GA or GA after the age of 24 months, no computer or internet access, or the child in question has a learning disability.

### Randomization

Randomization will be by minimisation in terms of year of age at time of recruitment (age groups: 5, 6, or 7) and gender. This will be performed centrally at the Biostatistics and Research Methods Centre of the Dental Institute, King’s College London. This will ensure not only that the proportion of 5, 6, and 7-year-old boys and girls will be balanced each week following each recruitment session, but also that the unblinded researcher cannot bias participant group allocation. The procedure for this has already been detailed in the pilot study.

### Intervention

The intervention is an online GA-coping computer game. It is approximately 10 screens in length and takes about 15 minutes to play. It has a cartoon family, embedded video and contains preparatory information, role modelling, fasting instructions, and oral health messages and is specific to the dental surgery topic.

At the time of recruitment, each participant will be given the intervention pack as appropriate for their group allocation and advised to use it at home one week prior to the GA dental visit. In addition, both the study group and the hand-washing control group will be prompted once via email to use their online game at home. The reason for this prompt is to overcome the problem of a greater than two-week time lag between recruitment and GA dental visit due to service events (for example, if child is rescheduled due to having a cold). This type of child-coping intervention aimed at 5 to 7-year-old children is ideally used a week before the hospital visit to enable children of this age to assimilate the information.

All participants will be invited to use their intervention on the ward of the Day Surgery Unit at the time of the GA dental visit. All participants, irrespective of group, will be asked if they used their intervention at home. The researchers will be able to automatically verify usage of the GA-coping game since each will have a unique username. The website will also automatically monitor how often, how long and how far the participating families progress through the game.

The themes of the control interventions were selected for relevance; the colouring book was about healthy food choices and the computer game was about personal hygiene and hand-washing. The control group computer game was further matched to the test intervention on specific characteristics such as length of time taken to play, and has a similar combination of cartoon and human features as the intervention. The interventions have been well matched and thought through by the child psychologist on the research team (CL) and all have some dental and hospital relevance, but only the intervention group has preparatory information specific to both general anaesthesia and surgical tooth extraction.

### Blinding

#### Participants

All participants will believe that they have been given some form of ‘intervention’ for home use. Irrespective of group allocation, the participants will be given identical packs to take home with them and advised to use them at home. Inside the blank control pack will be the normal patient information leaflet and a colouring book and inside the packs of the other two groups will be the logon information for the online games. The blank control group will be given nothing on the ward on the day of the GA visit and the other two groups will be given a laptop at their bedside with either the hand-washing or GA-coping game. All study patients will have curtains drawn around their beds which is common practice on the busy ward, especially when children are changing clothes. This will avoid contact between participants to avoid contamination and the unblinding of both participants and the blind data collector.

### Blinded observer (RA)

All of the key outcome measures on the day of the GA treatment visit will be collected by a second researcher (RA), blind to the group allocation. These include preoperative anxiety, acceptance of anaesthetic induction, details concerning the operative procedure (such as anaesthetic type), the anaesthetists’ view of patient compliance, service throughput timings, and the family’s satisfaction prior to discharge.

Only the research associate (CH) will be aware of the group allocation. The research associate will carry out the recruitment, consent, and randomization procedures, collect demographic information, and collect baseline measures including psychological wellbeing and dental anxiety (these will only be used to inform equivalence between the groups) at the time of recruitment. They will also deliver the intervention packs and conduct the 48 hour and 1 week follow-up telephone calls.

### DSU staff

The anaesthetist, operating dentist, and the operating theatre and recovery staff will be blinded to the group allocation. It is hoped that they will hardly be able to identify participants from non-participants.

### Statistician

The group allocation will remain concealed until after the statistical analysis has been concluded. The unblinded researcher (CH) will check whether families have used the GA-coping game at home. After this analysis is completed the automated user logs will be further investigated; since the URL logon for each study group family matches their study ID.

### Measures

The primary outcome measures are: (1) child cooperation and distress during the GA dental visit, on the ward before the procedure using the m-YPAS (time 1) (non-blinded researcher/observer reported) and the FIS child-reported to unblinded researcher [18,19]; (2) behaviour at anaesthetic induction by A-VAS score [16] and m-YPAS (time 2), reported by blinded researcher; (3) physical and psychological morbidity postoperatively using the Rutter Scale, Morbidity Checklist, and the Post Hospital Behaviour Questionnaire (unblinded-researcher recorded), parentally reported; (4) parent and child satisfaction using a 10 cm VAS (S-VAS) at the time of discharge from the Day Surgery Unit (parentally reported to the blinded observer) and the Treatment Evaluation Inventory at the 48 hour telephone call (parentally reported to the unblinded researcher); (5) service throughput will be measured using the duration of time in the induction room, duration of time in the recovery room, and duration of time on the ward before discharge (blinded-researcher recorded).

The secondary outcome measures are: the internet package usage by questioning about ease of access, acceptability, satisfaction- by parental reporting to the unblinded researcher at time of delivery of the allocated interventions, on the ward, and automatic recording via the internet.

The measures are detailed further:

At the time of recruitment the Rutter scale will test psychological well-being and status; this is a parental reporting tool but the unblinded researcher will be on hand to assist the parent with the questions if they have difficulty with literacy [23]. The child self-report of dental anxiety using MCDAs [20] supported by the Facial Image Scale (FIS) to aid comprehension [21], will be scored by the child themselves but supported by the unblinded researcher and the parent.

At the time of the GA treatment visit on the DSU ward before being taken into the induction room all child participants will complete the FIS (FIS-ward) to self-report situational anxiety and the Bieri Scale to record pain (toothache) [26] and will be asked about their usage of the intervention. These will be recorded by the unblinded researcher since these families might be using their allocated intervention at this time. The child’s behaviour will be observed by the blinded observer and scored using the modified Yale Preoperative Anxiety Scale (m-YPAS-time 1) approximately 10 minutes before they are due to leave the ward and be taken into the anaesthetic induction room.

The behaviour at anaesthetic induction will be video recorded and the observed behaviour (acceptance of anaesthetic induction via inhalation) scored by the blinded researcher using m-YPAS(-time 2) and a 10 cm Visual Analogue Scale (A-VAS) rounded to the nearest to 0.5; the video recordings will enable future intra-rater reliability and reproducibility testing on the A-VAS [13,17,18].

During the surgical procedure the blinded observer will record the number of teeth extracted, the dose of local anaesthetic administered (routinely given to aid haemostasis), and the dose, preparation and route of analgesics administered (usually paracetamol or NSAIDs rather than opioids).

Service throughput will be reported in respect to the duration of time in the anaesthetic induction room, the duration of time in the recovery room, and the duration of time on the ward prior to discharge. These times are routinely recorded on the computer system at King’s College Hospital by the DSU staff for use in the generation of theatre utilisation data; the data will be recorded and accuracy of the recorded times will be verified by the blinded observer.

In the recovery room, children’s observed behaviour will be scored using a 10 cm Visual Analogue Scale, to differentiate this from the other visual analogue scales used in the study this will be termed R-VAS and will be scored by the blinded researcher.

At the time of discharge, parents will be asked to rate their satisfaction with the preparatory information by the blinded researcher using a series of questions scored using a further 10 cm VAS (S-VAS).

AT 48 hours following the GA, and again one week later, parents will be contacted by the unblinded researcher by telephone in the evening and asked to rate their child’s behaviour and psychological wellbeing using the Rutter Scale [25] and asked to report on morbidity using a Morbidity Checklist (MC) and the Post Hospital Behaviour Questionnaire (PHBQ) [27]. They will also be invited to score their satisfaction with both the preparatory information and the service they received using the Treatment Evaluation Inventory (TEI) [23] and the child will be invited to self-report anxiety using the FIS [21]. Further demographic information will be asked at the one week data capture time point (parent age, highest education qualification, who lives in the house with the child, and whether the parent is a single parent or parents with another person) since it preserves family privacy and is remote from the stressful hospital visits. The family will also be invited to comment in their own words about their satisfaction and express their views on the preparation they received and the service that they experienced. Therefore, the group allocation will be unconcealed to the non-blinded observer.

For the web-based GA-coping game study group, participants’ usage will be captured automatically each time they access the site. Each unique website URL matches the family study ID. These logs will be analysed by one of the grant applicants (PAR) and the grant statistician (ND) after the RCT data has been analysed in full and the code broken.

### Intra-observer agreement of VAS at induction

Based on a random selection of 36 cases from RCT recruits, the A-VAS measure was rescored by the blinded observer (RA) using direct and video evaluation and confirmed an intra-class correlation coefficient of 87% with a 95% confidence interval of between 79 and 95%. This indicates from very good to excellent intra-observer agreement.

### Power calculation

Based on acceptance of anaesthetic induction measured using a 10 cm Visual Analogue Scale (A-VAS), using results from the Phase II study [13] for a statistical power of 90%, and for a difference of 2 cm on the Visual Analogue Scale (SD = 3.1) at the time of anaesthetic induction (an effect size of 0.65), 53 children will be required in each group. We have chosen this measure since it matches the Phase II RCT and has been well used and validated in this setting [15-17].

### Justification of sample size

We anticipate a dropout rate in the region of 20% and as such, a target group of 70 children for each of the groups is appropriate. Moreover, our previous work has shown that up to 30% of children referred for dental GA might have undergone a GA previously [13,28], and we expect a further 20% will refuse to participate due to concerns about filming, so we anticipate that we may have to approach as many as 300 potential recruits to achieve our target of 210 children.

### Statistical analysis

Intention-to-treat methodology will be employed. Descriptive statistical summaries for all explanatory variables at baseline will be provided, overall and by study group. Analysis of Variance and Chi-square tests will be used to highlight any significant imbalance between groups. The GA-coping game’s effect on the key primary outcome measures and the 10 cm VAS and the m-YPAS for child behaviour at anaesthetic induction, will be compared with the two controls and will be modelled using linear or ordinal regressions as appropriate. Multivariate models will be fitted so that any confounding effect is assessed and adjusted for if necessary.

## Discussion

Preparatory information benefits patients; de Freitas stated that online games are ‘applications using the characteristics of video and computer games to create engaging and immersive learning experiences for delivering specified learning goals, outcomes and experiences’ [29].

This study will be the first to test whether an interactive online GA-coping cartoon with an embedded video helps families to better, and more conveniently, prepare a child for surgery. We intend to focus on child dental patients because this is the most common group referred for general anaesthesia in the UK, yet it has the least ‘user friendly’ care pathway, it avoids sample variation in respect to anaesthetic method and surgery type, and is the service that will benefit most by reduction in family and staff stress and by resultant increase in throughput. However, in future, package access can be easily expanded UK-wide and the information gained used to develop further resources for children and adults who require other medical procedures such as blood tests.

We have based the sample size calculation on the blinded observer’s Visual Analogue Scale score at the actual moment of anaesthetic induction (A-VAS). Not only was this measure used in the Phase II RCT but it is also well validated for this purpose [16]. Moreover, the blinded researcher used the pilot study to train and calibrate this measure to avoid ‘ceiling’ effects and ensure reproducibility and reliability in the Phase III trial.

We also plan to use a 10 cm VAS (S-VAS) to measure family satisfaction with the preparatory information they received and with the service overall as they leave the hospital ward. The original plan was to use the TEI at this time, however, the pilot study showed that the families were tired and wanted to get back home at discharge time and were not keen to fully engage in a scoring measure that took longer and further delayed their departure. Therefore, we will use a simpler measure at time of discharge and score the TEI only at the telephone follow-up capture. Regarding the validity of these satisfaction measures, the use of a 10 cm VAS is superior to a Lickert scale when used as a single measure of patient satisfaction. It has also been validated for usage in this way and in a similar postoperative time period in adults undergoing arthroscopy [30]. We plan to ask the parents to report on the family satisfaction with the preparatory information that they received as well as the overall treatment experience using this 10 cm VAS (S-VAS). The TEI is a well-validated measure and has been slightly modified for the proposed study to clarify that it is scoring satisfaction with the preparatory information that was provided as well as the overall satisfaction with the service that the families rather than the dental treatment alone. The original author and validator of the TEI is one of the research team (TN) [21].

The removal of the ward discharge TEI capture point so that the only recording will be during a follow-up telephone call will mean that this measure will be collected by the unblinded researcher. However, given that the families are being asked specifically for their views about their allocated intervention, it would have been impossible to ensure continued researcher blinding in any case. Moreover, the TEI is a self-report measure. Nevertheless, this will have to be taken into account in the interpretation of the Phase III RCT results.

The FIS is well-validated for use, especially in dental settings. It is easy for a child to score this with the support of their parent or carer. It is a sheet of paper with symbols of faces [21]. It is the unblinded researcher who will have to collect this measure from the child on the ward since the child may be using the intervention or discussing it at that time point. Therefore, the researchers are aware that results obtained using this measure must be treated with caution. Indeed, the anxiety, psychological, and demographic data at the time of recruitment, family information such as maternal education and reported toothache on the day of the GA visit, as well as details of the anaesthesia, surgery and recovery, and analgesia will be used to report both equivalence between the groups and explain the patient journey so that the key results can be interpreted in context.

In paediatric dental anxiety studies it has been recommended that multiple measures are used; the researchers have adopted this principal for use in the Phase III RCT [31]. As such, we plan to use child and parent reporting as well as direct observation to record responses to the intervention. There are three key time points: on the ward before the anaesthesia, at the time of the anaesthetic induction, and then later at home. If this was a drug trial it would be easy to keep the data collection on the ward blind throughout, but since this is a computer intervention and the laptop computer will be open on the child’s bed (albeit with the curtains closed), the likelihood of unblinding is high. It is partly for this reason that such a comprehensive pilot study was needed.

Furthermore, ideally, the anaesthetists should be allowed to talk about the intervention with the child at the time of induction as it will help with the patient-doctor communication and engagement. Thus it might help with the anaesthetic induction and give a score in favour of the intervention. However, the researchers believe that blind data collection at this point is vital and so the hospital staff have been asked not to unblind RA.

The researchers have anticipated that data captured at the telephone follow-ups might be influenced by the Hawthorne effect since the families will already have met the researcher (CH) twice - once at recruitment and again on the ward [19]. CH is a trained researcher and is aware of this and of potential bias and will seek to minimise this. The pilot study helped to develop the script for the telephone dialogue so that the questioning of the families was kept as standard as possible for each telephone interview. Indeed, the principal investigator (MTH) in a previous RCT in this patient group noted that the personal engagement through frequent contact with study researchers might have had an adverse influence on the study outcome [28]. It is for this reason that the blinded researcher (RA) will not be introduced to the families and will collect mainly observational data.

## Trial status

The trial closed on 4^th^ June 2014, 185 participants were recruited, the attrition rate was less than anticipated and so the target sample number of 53 was met in each group more readily than anticipated.

## Abbreviations

A-VAS: Anaesthetic induction 10 cm VAS; DSU: Day Surgery Unit; FIS-ward: Facial Image Scale used by child to self-report anxiety on the ward; FIS-48: Facial Image Scale used by child to self-report anxiety at the 48 hour follow-up call; FIS-7: Facial Image Scale used by child to self-report anxiety at the 1 week follow-up call; GA: General anaesthesia; KCH: King’s College Hospital NHS Foundation Trust; MC: Morbidity Checklist used here to record both 48 hr and 1 week morbidity; MCDAs: Modified Child Dental Anxiety score; m-YPAS: modified Yale Preoperative Anxiety Scale; PHBQ: Post Hospital Behaviour Questionnaire used here to record 48 hr and 1 week morbidity; satis-VAS: 10 cm VAS at discharge to measure family satisfaction with the preparatory information; TEI: Treatment Evaluation Inventory used to rate the overall family view of the service they received and their satisfaction with the preparatory information.

## Competing interests

The authors declare that they have no competing interests.

## Authors’ contributions

MTH conceived the study, led its design and coordination, is project manager, and drafted this manuscript. JTN provided trial expertise. CH, the research associate, prepared materials for this article, organized the feasibility studies for the RCT that refined the methodology and produced the Standard Operating Procedures (SOPs) and led the training and calibration of the blind data measures, she contributes to the daily trial management and patient selection and recruitment. RA is the blinded data collector and participated in the feasibility studies and production of the SOPs. ND did the statistical analysis plan and contributed to the overall methodology of the study. PR provided input from an IT perspective. CL provided pediatric psychology expertise and played a key role in computer package design along with MTH and JTN. CL also provided qualitative expertise and oversaw all aspects of the qualitative part of the study. All authors have read and contributed to the manuscript.
